# Achieving Large-Capability Adsorption of Hg^0^ in Wet Scrubbing by Defect-Rich Colloidal Copper Sulfides under High-SO_2_ Atmosphere

**DOI:** 10.3390/ma16083157

**Published:** 2023-04-17

**Authors:** Xiaofeng Xie, Hao Chen, Xudong Liu, Kaisong Xiang, Hui Liu

**Affiliations:** 1 School of Metallurgy and Environment, Central South University, Changsha 410083, China; 2 State Key Laboratory of Advanced Metallurgy for Non-Ferrous Metals, Changsha 410083, China; 3 Chinese National Engineering Research Center for Control & Treatment of Heavy Metal Pollution, Changsha 410083, China

**Keywords:** mercury, copper sulfides, adsorption, wet scrubbing, high-SO_2_ flue gas

## Abstract

This paper reports on a novel method to remove Hg^0^ in the wet scrubbing process using defect-rich colloidal copper sulfides for reducing mercury emissions from non-ferrous smelting flue gas. Unexpectedly, it migrated the negative effect of SO_2_ on mercury removal performance, while also enhancing Hg^0^ adsorption. Colloidal copper sulfides demonstrated the superior Hg^0^ adsorption rate of 306.9 μg·g^−1^·min^−1^ under 6% SO_2_ + 6% O_2_ atmosphere with a removal efficiency of 99.1%, and the highest-ever Hg^0^ adsorption capacity of 736.5 mg·g^−1^, which was 277% higher than all other reported metal sulfides. The Cu and S sites transformation results reveal that SO_2_ could transform the tri-coordinate S sites into S_2_^2−^ on copper sulfides surfaces, while O_2_ regenerated Cu^2+^ via the oxidation of Cu^+^. The S_2_^2−^ and Cu^2+^ sites enhanced Hg^0^ oxidation, and the Hg^2+^ could strongly bind with tri-coordinate S sites. This study provides an effective strategy to achieve large-capability adsorption of Hg^0^ from non-ferrous smelting flue gas.

## 1. Introduction

Mercury has been a contaminant of global concern due to its toxicity, persistence, and bioaccumulation since the implementation of *the Minamata Convention* on August, 2017 [1]. Among anthropogenic sources, the non-ferrous smelting sector is a major contributor, accounting for 14.9% of all worldwide emissions, as reported in *the Global Mercury Assessment Report 2018* [2,3]. Therefore, it is urgent to efficiently remove Hg^0^ from non-ferrous smelting flue gas to abate the significant challenge of global mercury pollution control.

There are various forms of mercury in non-ferrous smelting flue gas, including elemental mercury (Hg^0^), oxidized mercury (Hg^2+^), and particulate mercury (Hg^p^) [4,5,6]. In the existing flue gas purification system, Hg^p^ would be captured by the electrostatic precipitator (ESP) [7,8,9]. Hg^2+^ has strong water solubility and can be removed by wet scrubber, whereas the capture of Hg^0^ is difficult due to its strong stability, high volatility, and low solubility [10]. Wet methods for Hg^0^ removal promote the conversion of Hg^0^ to Hg^2+^, such as the Boliden–Norzink and advanced oxidation process, thereby improving mercury removal efficiency [11,12]. Hence, oxidation is considered as a vital way for Hg^0^ removal in the wet scrubbing of non-ferrous smelting flue gas [13].

Oxidative demercurization remains a major bottleneck in the application of wet scrubbing from non-ferrous smelting flue gas [14,15]. There is a large amount of reductive SO_2_ (>6% vol) in the flue gas, which is thousands of times higher than Hg^0^ concentration [16,17]. Selective oxidation of Hg^0^ poses a great challenge since the standard oxidation potential of Hg^0^ (0.85 V) is higher than that of SO_2_ (0.17 V) [18,19]. Consequently, SO_2_ is preferentially oxidized over Hg^0^, bringing about the depletion of oxidants and making it difficult to oxidize Hg^0^. Therefore, the efficient oxidation of Hg^0^ from high-SO_2_ flue gas is key to overcoming the bottleneck of Hg^0^ removal in wet scrubbing.

Transition metal sulfides (TMSs) have become a hot material for Hg^0^ capture due to the strong affinity of sulfur sites with Hg^0^ [20,21,22]. Previous studies had shown that SO_2_ could react with defective S^2−^ on TMSs to generate highly active S_n_^2−^, which might promote Hg^0^ oxidation [23]. O_2_ dissolves into aqueous phase and forms dissolved oxygen, which has a high oxidation activity due to hydrogen bonding and Van Der Waals force with water molecules, providing an oxidative environment for TMSs to capture Hg^0^ [24]. Therefore, if TMSs are added to the wet scrubber, it is expected to eliminate the negative impact of SO_2_ on the removal of Hg^0^, which could enhance the ability of TMSs to oxidize mercury, thereby greatly improving the adsorption capacity of mercury. However, the mechanism of SO_2_ and O_2_ in wet scrubbing for mercury capture by TMSs has not been reported.

Based on the above considerations, this paper involves a novel method of using metal sulfides in the wet scrubbing process for large-capability adsorption of Hg^0^ from high-SO_2_ flue gas. The optimal mercury adsorbent was prepared by sulfide precipitation and optimized systematically. The morphology and structure of the adsorbents were characterized. The excellent Hg^0^ removal performance of colloidal copper sulfides (c-CuS) was examined in the flue gas wet scrubber. The effects of SO_2_ and O_2_ on Hg^0^ removal by c-CuS were investigated, and the mechanism of the activation of oxidizing sites (S_2_^2−^ and Cu^2+^) by SO_2_ and O_2_ was proposed after identifying the structural changes of c-CuS under different atmospheres.

## 2. Experimental Section

### 2.1. Chemicals and Reagents

The chemicals in analytical grade were used in this study, including copper chloride (CuCl_2_), copper nitrate (CuNO_3_·6H_2_O), Copper sulfate (CuSO_4_·5H_2_O), sodium sulfide (NaS·9H_2_O), sodium hydroxide (NaOH), sodium chloride (NaCl), and nitric acid (HNO_3_). They were purchased from Sinopharm Chemical Reagent Co., Ltd. (Shanghai, China) All the agents were directly used without any further purification. Ultrapure water was used in all experiments unless otherwise stated.

### 2.2. Synthesis of TMSs

TMSs suspensions were synthesized by the double-jet liquid-phase sulfidation precipitation method. Simply, 50 mL of a 10 mmol·L^−1^ metal chloride solution and an equal volume of 10 mmol·L^−1^ Na_2_S·9H_2_O solution were added simultaneously to the beaker at a speed of 1.2 mL min^−1^. These solutions were mixed by stirring at a speed of 380 rpm for 0.5 h, and then TMSs suspensions were obtained. c-CuS was synthesized using a single-jet liquid-phase sulfidation method. Then, 50 mL of Na_2_S·9H_2_O was rapidly added into a CuCl_2_ solution of 1.0 mmol·L^−1^, and then they were mixed by stirring. The experimental investigation on raw material concentration and ratio was undertaken to judiciously optimize the synthesis conditions.

### 2.3. Sample Characterization

These suspensions and colloids were filtered with nanopore filter membranes. The filtered residues were washed three times with ultrapure water and then dehydrated at −89 °C in a vacuum freeze-dryer for 8 h. The obtained filtered residues of TMSs suspensions and c-CuS were analyzed for crystalline structure by X-ray diffraction (XRD, Empyrean 2, PANalytic, Malvern, UK) with Cu-Kα radiation. High-resolution transmission electron microscopy (HRTEM, Titan G260-300, FEI, Lausanne, Switzerland) was used to observe the morphology of c-CuS particles. The scanning electron microscope (SEM, JSM-6360LV, Jeol, Tokyo, Japan) of c-CuS was characterized at the accelerating voltage of 200 kV after splashing a thin Au layer. The structure information of samples under different atmospheres was characterized by X-ray photoelectron spectroscopy (XPS, Escalab 250 Xi, Thermo Fisher Scientific, Waltham, MA, USA). The ultimate vacuum degree of the sample analysis room was 5 × 10^−7^ Pa, and the Al Kα X-ray was used as the excitation source with a power of 16 mA × 12.5 kV. Correction was made using C ls = 284.6 eV as the internal standard for electron binding energy. The fingerprint and structural properties of c-CuS were analyzed by three-dimensional excitation–emission matrix (3D-EEM, F7000, Hitachi, Ibaraki, Japan) fluorescence spectroscopy. 3D-EEM spectra were generated by scanning in the range of 200 nm to 600 nm, while excitation and emission sampling wavelengths were 10 nm, and the scanning speed was 12,000 nm·min^−1^. Finally, temperature-programmed desorption (TPD) was employed to identify the form of mercury species adsorbed on c-CuS. The TPD tests were carried out under pure nitrogen at the flow rate of 500 mL·min^–1^ from 50 °C to 600 °C at a rate of 5 °C·min^−1^.

### 2.4. Hg^0^ Removal Test

An experiment was conducted in a bubbling reactor to remove Hg^0^. Pure N_2_ was used as the carrier gas for the Hg^0^ generator (VICI Metronics) with a flow rate of 200 mL·min^−1^. The simulated flue gas (SFG) containing N_2_, SO_2_, and O_2_ at a flow rate of 300 mL·min^−1^ was mixed with the Hg^0^ carrier gas. The mixture gas was then blown into a bubbling reactor containing the scrubbing solution. Then, the SFG was treated with 5 mol·L^−1^ NaOH and quartz wool before the mercury detection to reduce the influence of SO_2_ and water vapor. The mercury analyzer (RA-915M, Lumex Zeeman) was used to monitor the outlet Hg^0^ concentration. All data were obtained by averaging three measurement results. Finally, the remaining Hg^0^ in the tail gas was absorbed by KMnO_4_ solution and activated carbon. The operating conditions in the experiment are listed in Table S1. The Hg^0^ removal efficiency and adsorption capacity were calculated according to Equations (1) and (2), respectively.

(1) $$\eta =\frac{C_{in}-C_{out}}{C_{in}}\times 100\%$$

(2) $$Q_{t}=1/M\times \int_{t1}^{t2}(C_{inlet}-C_{outlet})∆t\times u$$

where η (%) represents Hg^0^ removal efficiency, and *C_in_* and *C_out_* (μg·m^−3^) are the Hg^0^ concentration at the inlet and outlet of the experimental system, respectively. $Q_{t}$ (mg·g^−1^) is the adsorption capacity of Hg^0^, *M* (mg) is the mass of adsorbent, *t*_1_ and *t*_2_ (min) are the start and end times, Δ*t* (min) is the scrubbing time, and *u* is the gas flow rate (m^3^·min^−1^).

### 2.5. DFT Calculation

Perdew–Burke-Ernzerhof (PBE0) functional was used to characterize the exchange-correlation effects in the density functional theory (DFT) by CP2K [25,26]. The CuS (110) system was simulated using an orthorhombic box with dimensions of 16.44 × 13.15 × 34.49 Å3, and the dipole correction technique was applied in the XY orientation. BROYDEN-MIXING was used to optimize the geometries, while the DZVP-MOLOPT-SR-GTH basis set was used to describe the basis. A plane wave cutoff of 400 Ry was set, and Goedecker–Teter–Hutter (GTH) pseudopotentials were used to represent all electrons [27,28]. In addition, the D3 dispersion correction by Grimme et al. was employed, and the adsorption energy (*E_ad_*) was calculated using Equation (3), based on the optimized structure.

*E_ad_ = E_(Hg+CuS)_ − E_Hg_ − E_CuS_* (3)

where *E_H_*_g_ is the state energy of a free Hg atom, *E_CuS_* represents the total energy of the CuS of different configurations, and *E_(Hg+CuS)_* is the total energy of the Hg atoms being adsorbed on CuS.

## 3. Results and Discussion

### 3.1. Selection and Optimization of Transition Metal Sulfides for Hg^0^ Removal

**Figure 1:**
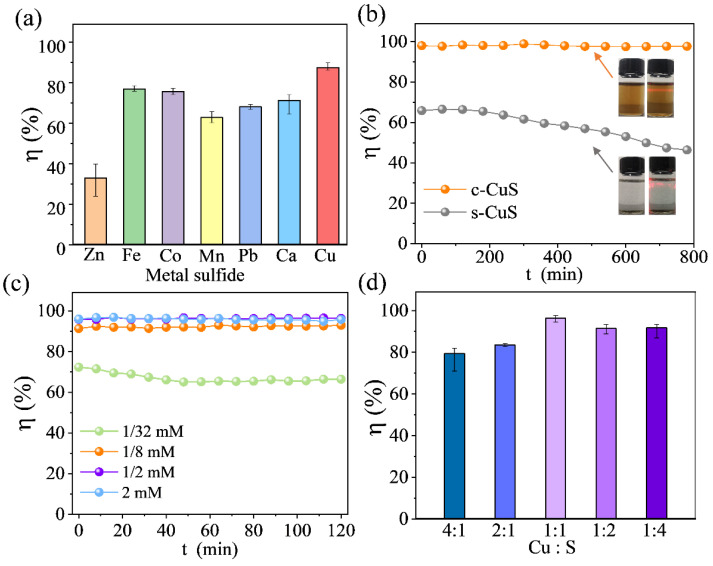
(**a**) Hg_0_ removal performances of different TMSs suspensions. (**b**) Long-time Hg_0_ removal performances of c-CuS and s-CuS. (**c**) The effect of raw material concentration on Hg^0^ removal. (**d**) The effect of Cu and S ratio on Hg^0^ removal (Experimental condition: solution volume = 80 mL, solution pH = 3.0, solution temperature = 20 °C, flue gas was pure N_2_, the inlet concentration of Hg^0^ was 200 µg·m^−3^).

Hg^0^ removal performance was evaluated by adding TMSs suspensions into a bubble reactor as the scrubbing solution. Figure 1a shows that TMSs suspensions had a good ability to capture Hg^0^ in wet scrubbing. Especially, copper sulfides suspension was the preferred Hg^0^ removal scrubbing agent, with the highest efficiency of 87.6%. Furthermore, the effect of regulating feeding methods on the dispersibility of detergent was investigated to improve the Hg^0^ removal performance of copper sulfides. Figure 1b displays that c-CuS prepared by single-jet was homodispersed in solution, and the Tyndall effect was evident, while the precipitation of suspensions copper sulfides (s-CuS) was visible. The removal efficiency gradually decreased from 65.8% to 46.4% due to the poor contact between gas and s-CuS, while the mercury removal efficiency of c-CuS could be maintained at 97.5 ~ 98.9%. Therefore, the c-CuS synthesized by single-jet has better Hg^0^ capture performance.

Optimization of the raw material concentration and ratio to synthesize copper sulfides is crucial for enhancing Hg^0^ removal. As shown in Figure 1c and Figure S1, when the concentration of c-CuS was 1/32 mmol·L^−1^, the efficiency of Hg^0^ removal was only 65% due to the insufficient content of active components in the solution. Increasing the concentration of CuCl_2_ and Na_2_S fourfold to synthesize c-CuS could immediately improve the removal efficiency of Hg^0^ to 93%. When the concentrations were optimized to 1/2 mmol·L^−1^ and 2 mmol·L^−1^, the efficiencies were higher than 96.3%. Unfortunately, a large amount of precipitation appeared in the solution of 2 mmol·L^−1^ concentration, which was not conducive to sufficient contact with Hg^0^. Finally, the Cu/S ratio of copper sulfides was optimized, as shown in Figure 1d. Copper sulfides had the best removal efficiency for Hg^0^, which was achieved by reaching 98.1% when the Cu:S ratio was 1:1. However, as shown in Figure S2, further increasing the S^2−^ ratio caused possible instability of colloid, which would have a negative effect on the Hg^0^ removal. In summary, the c-CuS was prepared with the concentration of 1/2 mmol·L^−1^ and the Cu:S raw material ratio of 1:1, which was the optimal mercury capture agent.

### 3.2. Structural Characterizations of c-CuS

**Figure 2:**
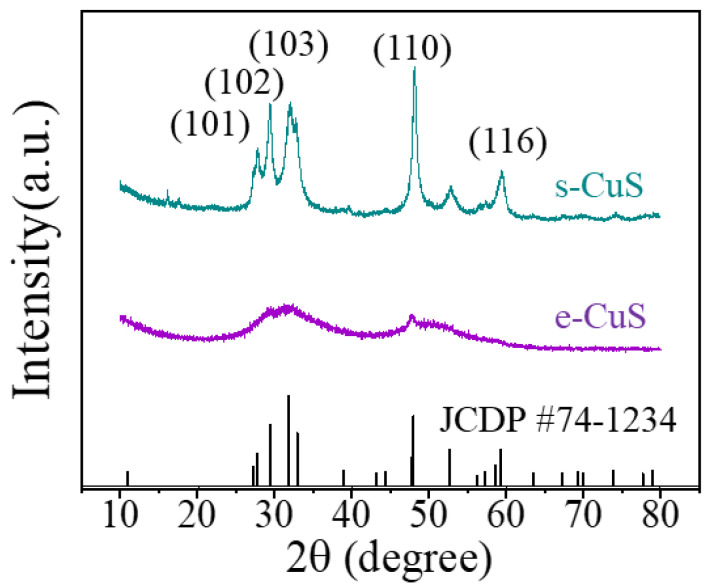
The XRD patterns of c-CuS and s-CuS.

Figure 2 shows the XRD patterns of the obtained c-CuS and s-CuS filter residues. The phase was confirmed to be Cu_4_(S_2_)_2_(CuS)_2_ (JCPDS card NO. 74-1234), with overlapping diffraction peaks that could be divided into lattice planes such as (101), (102), (103), (110), and (116) [29]. The diffraction peaks of s-CuS were stronger and sharper, indicating a better crystallinity. According to Equation (4), the crystallinity of s-CuS was calculated to be 70.9%. Surprisingly, the crystallinity of the c-CuS sample was only about 3.4% with severely broadened diffraction peaks. It indicated that c-CuS might contain abundant defects, which was beneficial for exposing more active adsorption sites to capture Hg^0^ [15,30].

(4) $$X_{c}=\frac{I_{c}}{I_{c}+I_{a}}\times 100\%$$

where *I*_c_ is the crystal diffraction intensity and *I*_a_ is the amorphous scattering intensity.

**Figure 3:**
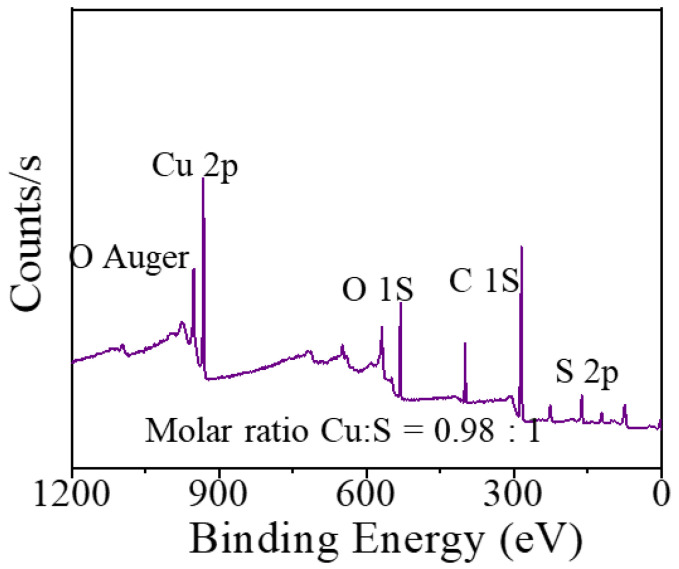
The XPS full spectra survey of c-CuS.

**Figure 4:**
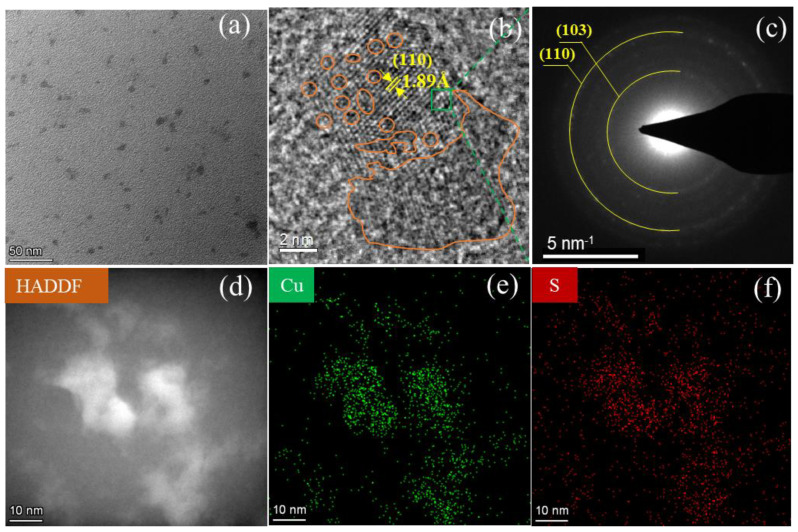
(**a**) TEM image of c-CuS. (**b**) HRTEM image of c-CuS. (**c**) SAED image of c-CuS. (**d**) HADDF image of c-CuS. (**e**) Cu and (**f**) S element mapping images of c-CuS (Prepared condition of c-CuS was the concentration of 1/2 mmol·L^−1^ and the Cu:S raw material ratio of 1:1 by using a single-jet liquid-phase sulfidation method).

Figure 3 shows the XPS full spectrum of c-CuS. The peak analysis result indicated that the Cu:S atomic ratio was 0.98:1 on the surface of c-CuS filter residue (the O, C peak belongs to the peak of carrier sheet). The mismatched stoichiometric ratio suggested that rich sulfur sites were exposed. Figure 4a shows that c-CuS was composed of near-spherical nanoparticles with the diameter of about 8.0 nm, similar to those of SEM in Figure S3. HRTEM images in Figure 4b display many orange-marked vacancies and amorphous regions in the lattice, indicating that rich defect that could provide more active sites for mercury capture. The measured lattice spacing of 1.89 nm was consistent with the (110) plane of c-CuS. The broad diffraction rings in the selected-area electron diffraction (SAED) shown in Figure 4c further confirmed the existence of amorphous regions in c-CuS [31]. Figure 4d–f shows the high-angle annular dark-field image (HADDF), Cu element, and S element scanning images of c-CuS, respectively. It confirms that Cu and S were evenly distributed. In summary, c-CuS with abundant defect sites has the potential for high-activity mercury capture.

### 3.3. Hg^0^ Removal Performance of c-CuS under SO_2_ and O_2_ Atmospheres

**Figure 5:**
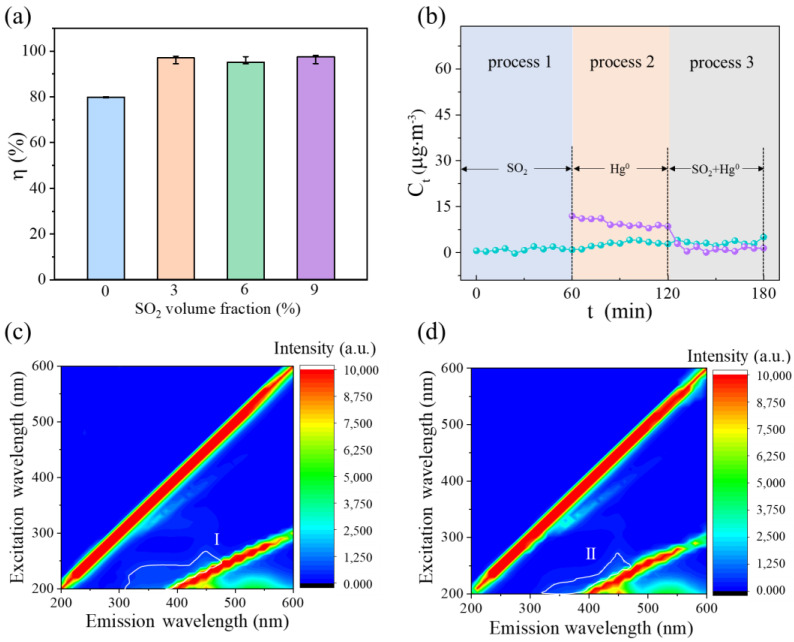
(**a**) The effect of SO_2_ on Hg^0^ removal efficiency. (**b**) Hg^0^ removal performances under SO_2_ on–off experiment. (**c**) The 3D-EEM fluorescence spectrum of c-CuS under pure N_2_. (**d**) The 3D-EEM fluorescence spectrum of c-CuS under SO_2_ (Experimental condition: solution volume = 10 mL, solution pH = 3.0, solution temperature = 20 °C, flue gas was 0 ~ 9% vol SO_2_ + N_2_, the inlet concentration of Hg^0^ was 200 µg·m^−3^).

SO_2_ is typically considered to be a negative effects gas that inhibits Hg^0^ removal in industrial flue gas [23,32]. SO_2_ resistance has a crucial impact on the mercury capture performance of adsorbents. SO_2_-contained simulated flue gas was inputted into a bubbling reactor with 10 mL of c-CuS to investigate the effect of SO_2_ on the Hg^0^ removal performance of c-CuS. As shown in Figure 5a, the removal efficiency was 80.1% in the absence of SO_2_. The efficiency significantly increased to 95.1 ~ 97.4% under 3 ~ 9% vol SO_2_. Furthermore, the performance of c-CuS for Hg^0^ removal was compared with and without SO_2_ pretreatment, as shown in Figure 5b. The outlet concentration of Hg^0^ was about 10 μg·m^−3^ after passing through c-CuS solution pretreated without SO_2_, but it decreased immediately to ~0 μg·m^−3^ after turning on SO_2_. When c-CuS solution was pretreated with SO_2_, the outlet Hg^0^ concentration was about 3 μg·m^−3^ after opening gas pipeline of Hg^0^. Meanwhile, the outlet concentration exhibited almost no fluctuation after supplying SO_2_ and Hg^0^ simultaneously. This might mean that SO_2_ did not occupy the adsorption sites, and SO_2_ could interact with c-CuS to enhance adsorption with Hg^0^. The 3D-EEM was used to compare the fluorescence fingerprint spectra of c-CuS under N_2_ + 6% vol SO_2_ atmospheres. As shown in Figure 5c,d, the region II area of the sample treated with SO_2_ was smaller than the region I area of c-CuS under N_2_ atmosphere, confirming that SO_2_ leads to the formation of more vacancies. The formation of vacancies meant that low-active S^2−^ had been converted to more active S_2_^2−^ [33,34]. The specific conversion process of activity sites will be further studied in the following.

**Figure 6:**
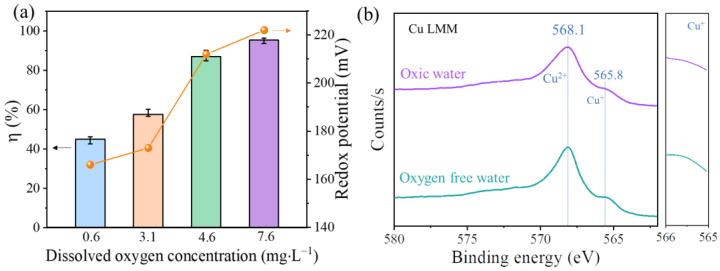
(**a**) The relationship of redox potential and Hg^0^ removal efficiency at varied dissolved O_2_ concentrations (Experimental condition: solution volume = 10 mL, solution pH = 3.0, solution temperature = 20 °C, flue gas was 0 ~ 9% vol O_2_ + N_2_, the inlet concentration of Hg^0^ was 200 µg·m^−3^). (**b**) XPS spectra of Cu LMM for c-CuS in oxygen-free water and oxic water.

In Figure S4, it could be observed that the capture of mercury was almost unaffected under an atmosphere of 3 ~ 9% vol O_2_. This was attributed to the fact that c-CuS solution could dissolve only 0.076 mmol·L^−1^ oxygen, which was much higher than the amount of mercury and much lower than the O_2_ proportion in the flue gas. Therefore, we conducted further investigations to explore the influence of dissolved oxygen on Hg^0^ oxidation. As displayed in Figure 6a, the dissolved oxygen concentration in c-CuS solution increased from 0.6 mg∙L^−1^ to 7.6 mg∙L^−1^. The redox potential of the c-CuS solution gradually increased from 166 mV to 222 mV, which meant enhancing the oxidation ability of the scrubbing solution. It led to a significant increase in the removal efficiency of mercury from 45.0% to 95.3%. As shown in Figure 6b, the results displayed that the Cu LMM auger spectral peaks included Cu^2+^ of binding energy at 568.1 eV and Cu^+^ at 565.8 eV [35]. The Cu^+^ spectral peak of c-CuS in oxygen-free water was visible, while it was weakened for c-CuS in oxic water, conversely indicating the oxidation of Cu^+^ to Cu^2+^ by O_2_. It could be inferred that dissolved oxygen in an aqueous solution could increase the Cu^2+^ content of c-CuS and strengthen the oxidation of Hg^0^.

**Figure 7:**
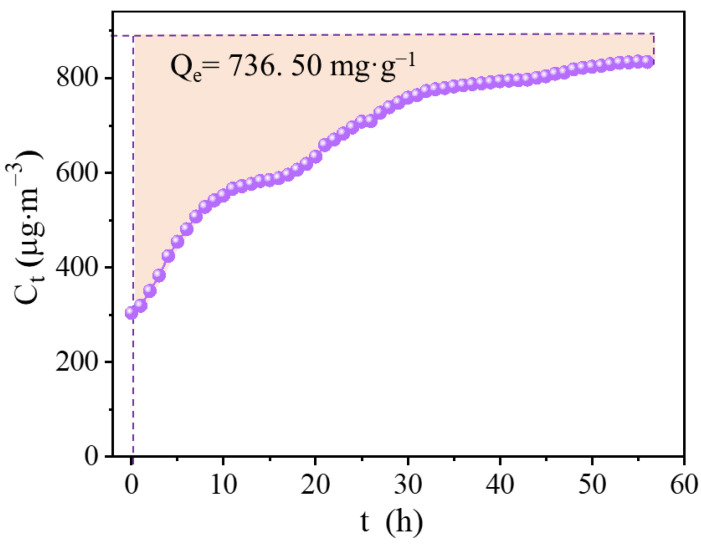
The adsorption breakthrough curve of c-CuS under 6% vol SO_2_+ 6% vol O_2_ (Experimental condition: solution volume = 10 mL, solution pH = 3.0, solution temperature = 20 °C, flue gas was 6% vol SO_2_ + 6% vol O_2_ + N_2_, the inlet concentration of Hg^0^ was 890 µg·m^−3^).

**Figure 8:**
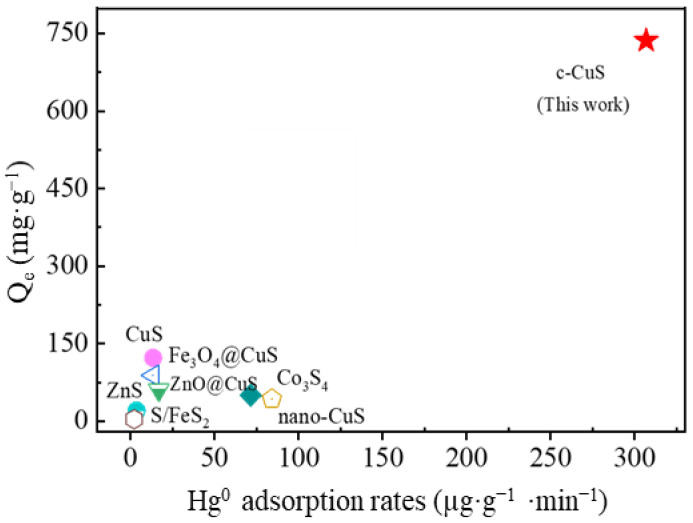
Comparison of adsorption capacity and rate with reported metal sulfide materials.

The Hg^0^ adsorption capacity and rate of c-CuS were investigated and calculated by penetration experiments. The prepared 10 mL c-CuS (containing 0.48 mg CuS particles) was input with 6% vol SO_2_ + 6% vol O_2_ flue gas containing an inlet Hg^0^ concentration of 890.0 µg·m^−3^. As shown in Figure 7, the outlet concentration of Hg^0^ gradually increased from 303.2 µg·m^−3^ to 835.5 µg·m^−3^ after 2400 min of scrubbing experiment. The Hg^0^ adsorption capacity of c-CuS was calculated to be 736.5 mg·g^−1^, and the average adsorption rate was 306.9 μg·g^−1^·min^−1^. Figure 8 and Table S2 demonstrate the comparison of the adsorption capacity and rate of c-CuS with previously reported metal sulfides. c-CuS has the highest adsorption capacity and rate among the reported sulfide adsorbents. Compared to all other adsorbents, the Hg^0^ adsorption capacity of c-CuS is exceptionally high, exceeding them by an impressive 277%, such as nano-CuS, Co_3_S_4_, S/FeS_2_, ZnS, and so on. Additionally, the Hg^0^ adsorption rate of c-CuS is much higher than that of other mineral sulfides, which is due to the abundant adsorption sites under the positive effect of SO_2_ and O_2_ [7,14,36,37,38,39,40].

### 3.4. Mechanism for Hg^0^ Adsorption

**Figure 9:**
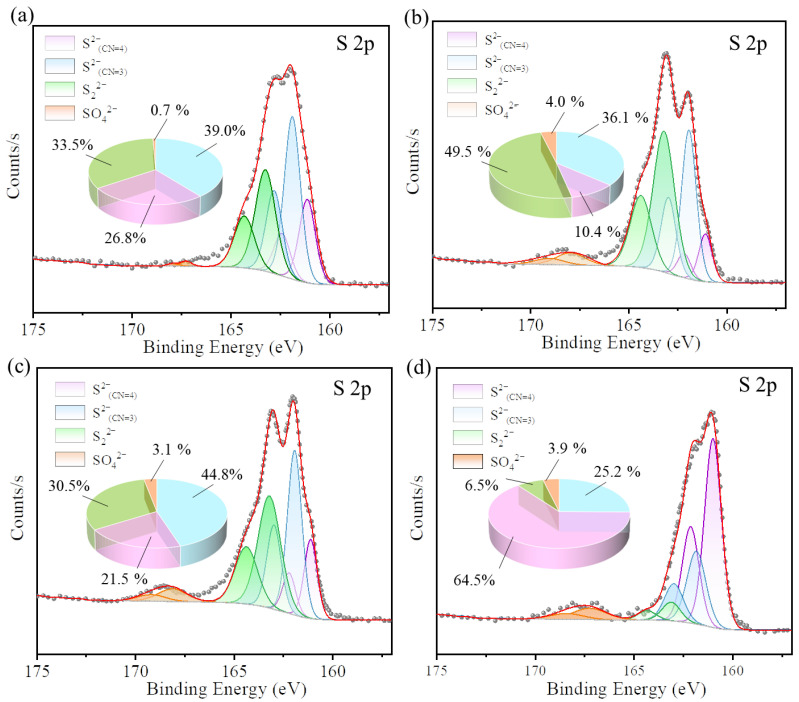
XPS spectra of S 2p for c-CuS under (**a**) N_2_, (**b**) 6% vol SO_2_, (**c**) 6% vol SO_2_ + 6% vol O_2_, (**d**) 6% vol SO_2_ + 6% vol O_2_ +Hg^0^ (Experimental condition: solution volume = 80 mL, solution pH = 3.0, solution temperature = 20 °C, the inlet concentration of Hg^0^ was 200 µg·m^−3^).

As mentioned above, SO_2_ and O_2_ have positive effects on c-CuS for Hg^0^ capture. To determine the mechanism of Hg^0^ adsorption under SO_2_ and O_2_ atmospheres, as shown in Figure 9, the XPS spectra of spent c-CuS samples were analyzed under different atmospheres. Shown in Figure 9a–d are the S 2p peaks of samples under N_2_, 6% vol SO_2_, 6% vol SO_2_ + 6% vol O_2_, and 6% vol SO_2_ + 6% vol O_2_ + Hg^0^, respectively. The forms of S on the c-CuS surface are multiple, such as tri-coordinated S^2−^_(CN=3)_, tetra-coordinated S^2−^_(CN=4)_, and sulfur–sulfur-coordinated S_2_^2−^ [23]. After peak fitting, the peaks at 161.1 eV and 162.2 eV were assigned to S^2−^_(CN=3)_, while the peaks at 161.9 eV and 163.0 eV were attributed to S^2−^_(CN=4)_. The peaks at 163.2 eV and 164.4 eV were attributed to S_2_^2−^, while the peaks at 167.9 eV and 168.9 eV belonged to SO_4_^2−^ [37,38,41].

**Figure 10:**
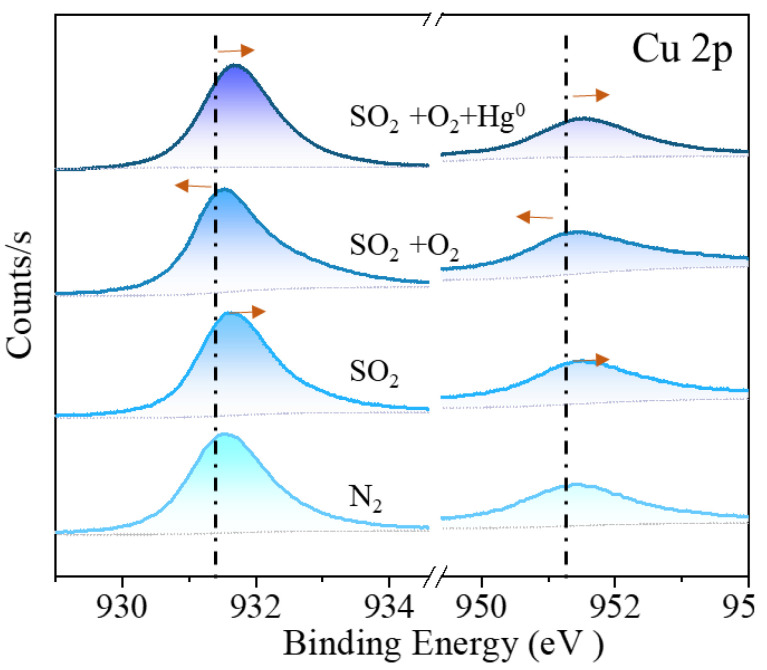
Cu 2p of spent c-CuS samples under pure N_2_, 6% vol SO_2_, 6% vol SO_2_ + 6% vol O_2_, and 6% vol SO_2_ + 6% vol O_2_ +Hg^0^.

**Figure 11:**
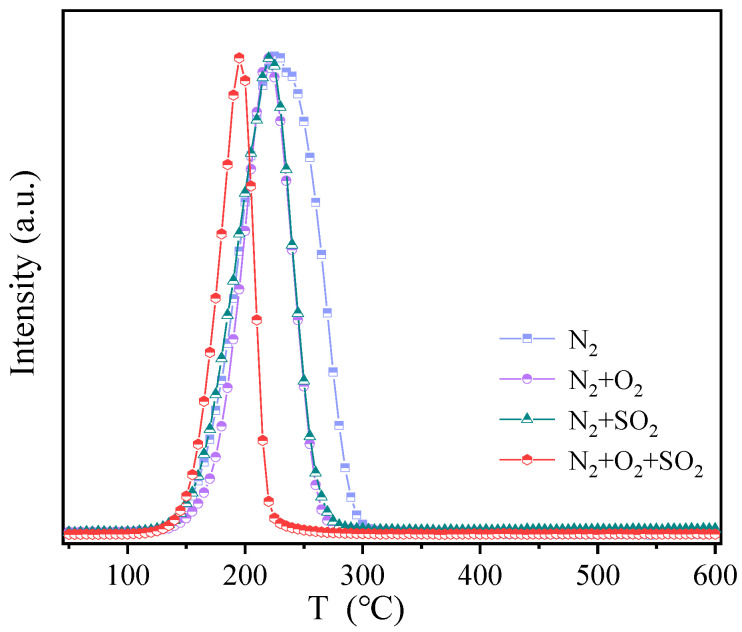
The TPD curves of spent samples after Hg^0^ adsorption under different atmospheres.

Under N_2_ atmosphere, the ratio of S and Cu total amount (S_t_, Cu_t_) on c-CuS was found to be 1.01:1, with S 2p consisting of 26.8% S^2−^_(CN=3)_, 39.0% S^2−^_(CN=4)_, 33.5% S_2_^2−^, and 0.7% SO_4_^2−^. Upon treatment with 6% vol SO_2_, the S_t_:Cu_t_ ratio increased to 1.79:1, with a decrease in S^2−^_(CN=3)_ content to 10.4%, an increase in S_2_^2−^ to 49.5%, and a slight change in other forms of S, indicating the formation of new S_2_^2−^ by the combination of S^2−^_(CN=3)_ with SO_2_. Additionally, the Cu 2p peak in Figure 10 shifted towards higher binding energy, indicating the reduction of a part of Cu^2+^ to Cu^+^ by SO_2_. Similarly, under 6% vol SO_2_ + 6% vol O_2_ conditions, S_t_:Cu_t_ decreased with the oxidation of Cu^+^ into Cu^2+^ by O_2_ and inhibition of S^2−^_(CN=4)_ combination with SO_2_ adsorbed on the c-CuS surface. The S^2−^_(CN=3)_ content decreased with an increase in S^2−^_(CN=4)_, which led to less S_2_^2−^. The further supply of Hg^0^ resulted in a decrease in the ratio of S_2_^2−^ and S^2−^_(CN=4)_, but an increase in S^2−^_(CN=3)_, indicating the transformation of S_2_^2−^ and S^2−^_(CN=4)_ into S^2−^_(CN=3)_ after capture of Hg^0^. The results of Cu 2p shifting towards higher binding energy confirmed that Cu^2+^ was also an active site for Hg^0^ oxidation. It suggests that S_2_^2−^ and Cu^2+^ serve as oxidation sites, while S^2−^_(CN=3)_ is the binding site with Hg after adsorption. The TPD results in Figure 11 indicated that the decomposition temperature of Hg on the c-CuS was about 195 ~ 220 °C, which suggested the presence of black HgS [38].

**Figure 12:**
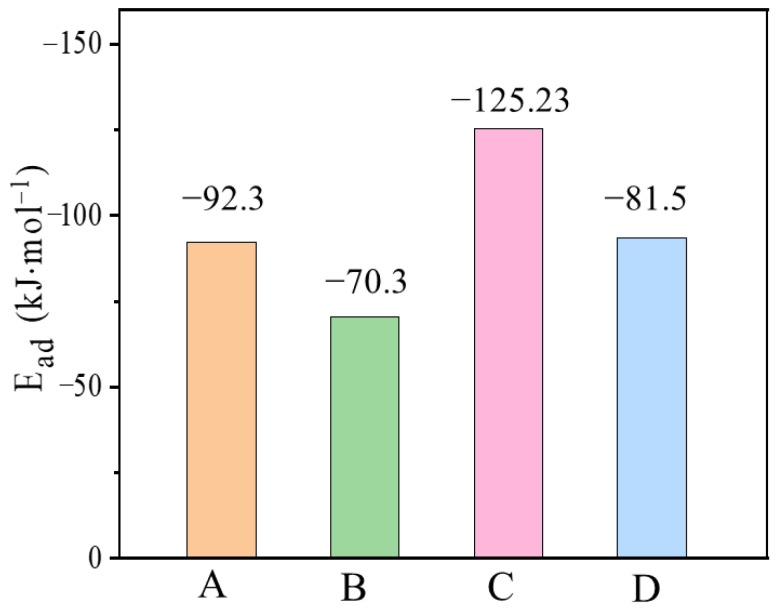
The Ead on CuS (110) of Hg^0^ with different sites (A: di-coordinated Cu, B: tri-coordinated Cu, C: tri-coordinated S, and D: tetra-coordinated S. The structural configurations shown in Figure S4).

The experimental results were inconclusive about the main binding site of the adsorbed mercury. Therefore, the E_ad_ at each site on the c-CuS (110) crystal was analyzed at the molecular level using DFT calculations. The result in Figure 12 indicated that the tri-coordinate sulfur sites had the highest adsorption energy of −125.23 kJ·mol^−1^. It means that the tri-coordinate sulfur site might be the main binding site for mercury, which is consistent with XPS results.

In summary, the mechanism for Hg^0^ capture under SO_2_ and O_2_ atmospheres could be described by Equations (5) ~ (10). After the adsorbing of Hg^0^ on the c-CuS surface, Cu^2+^ and S_2_^2−^ sites oxidized Hg^0^_ad_ to Hg^2+^ and binded with S^2−^_(CN=3)_ to form HgS. As described in Equations (9) and (10), SO_2_ and O_2_ respectively activate S^2−^_(CN=3)_ and Cu^+^ transformed into S_2_^2−^ and Cu^2+^ as Hg^0^ oxidation sites.

Hg^0^ → Hg^0^_ad_ (5)

Hg^0^_ad_ + 2Cu^2+^ − S^2−^_(CN=4)_ → Hg^2+^ + 2Cu^+^ − S^2−^_(CN=3)_ (6)

Hg^2+^ + S^2−^_(CN=3)_ → HgS (7)

Hg^0^_ad_ + S_2_^2−^ → HgS + S^2−^_(CN=3)_ (8)

4Cu^+^ + O_2_ + 4H^+^ → 4Cu^2+^ + 2H_2_O (9)

3S^2−^_(CN=3)_ + HSO_3_^−^+ 3H^+^ → 2S_2_^2−^ + 2H_2_O (10)

## 4. Conclusions

In this paper, the optimal conditions for preparing c-CuS with a Cu–S ratio of 1:1 and a concentration of 0.5 mmol·L^−1^ were synthesized by a double-jet liquid-phase sulfidation precipitation method. Defect-rich c-CuS with low crystallinity was used as a scrubbing agent to remove mercury from flue gas in wet scrubbing. It migrated the negative effect of SO_2_ on mercury removal performance, while also enhancing mercury capture. The Hg^0^ removal efficiency of c-CuS was 99.1% under 6% vol SO_2_ + 6% vol O_2_ atmosphere. The Hg^0^ adsorption capacity of c-CuS reached 736.5 mg·g^−1^, and the average adsorption rate was 306.9 μg·g^−1^·min^−1^, which was far better than other reported metal sulfides adsorbents. Based on structural characterization and DFT calculation, it was found that SO_2_ and O_2_ can enhance the formation of Cu^2+^ and S_2_^2−^ sites from Cu^+^ and S^2−^_(CN=3)_ to promote the oxidation of Hg^0^ to Hg^2+^, and then Hg^2+^ could strongly bind with S^2−^_(CN=3)_. However, reusability of the material should be developed in future studies.

## Data Availability

Not applicable.

## Supplementary Materials

The following supporting information can be downloaded at https://www.mdpi.com/article/10.3390/ma16083157/s1: Figure S1. Pictures of c-CuS prepared at different concentrations. Figure S2. (a) The pictures of c-CuS prepared with different Cu:S ratios. (b) Their phenomenon of Tyndall Effect. Figure S3. The SEM pictures of c-CuS. Figure S4. The effect of O_2_. Figure S5. The structure of configurations A, B, C, and D after mercury adsorption. Table S1. List of experimental conditions for Hg^0^ capture. Table S2. Hg^0^ adsorption capacities and rates of different sulfide sorbents. References [36,38,39,42,43,44,45,46,47,48,49] are cited in the supplementary materials.
