# Supplementary material for: Achieving Large-Capability Adsorption of Hg0 in Wet Scrubbing by Defect-Rich Colloidal Copper Sulfides under High-SO2 Atmosphere
Source: Materials (Basel). 2023 Apr 17;16(8):3157. doi: 10.3390/ma16083157 (PMC10144309; doi:10.3390/ma16083157)
Supplement: Supplementary file 1 [file materials-16-03157-s001.zip › materials-2340213-supplementary.pdf]

Achieving Large-capability Adsorption of Hg^0 in Wet Scrubbing by Defect-rich Colloidal Copper Sulfides under High-SO_2 Atmosphere

Xiaofeng Xie [1], Hao Chen [1], Xudong Liu [1], Kaisong Xiang [1,2,3,*], Hui Liu [1,2,3,*]

[1] School of Metallurgy and Environment, Central South University, Changsha 410083, China

[2] State Key Laboratory of Advanced Metallurgy for Non-ferrous Metals, Changsha 410083, China

[3] Chinese National Engineering Research Center for Control & Treatment of Heavy Metal Pollution, Changsha 410083, China

* Corresponding address:

Hui Liu: leolau@csu.edu.cn

Kaisong Xiang: xiangkaisong@hotmail.com

FIGURES:

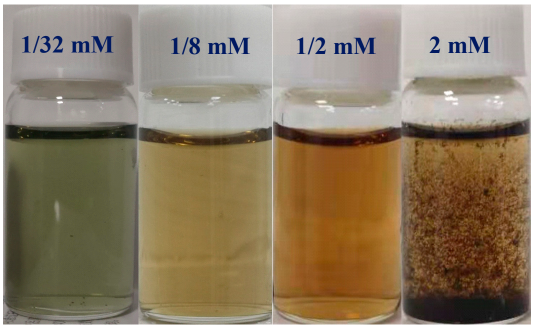

Figure S1. Pictures of c-CuS prepared at different concentration.

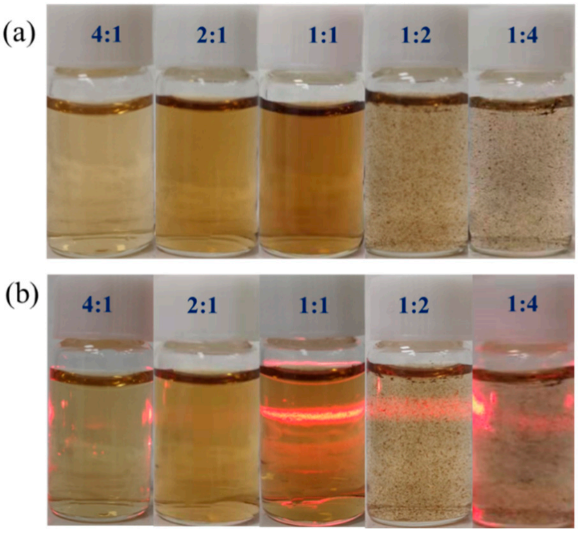

Figure S2. (a) The pictures of c-CuS prepared with different Cu: S ratio. (b) Their phenomenon of Tyndall Effect.

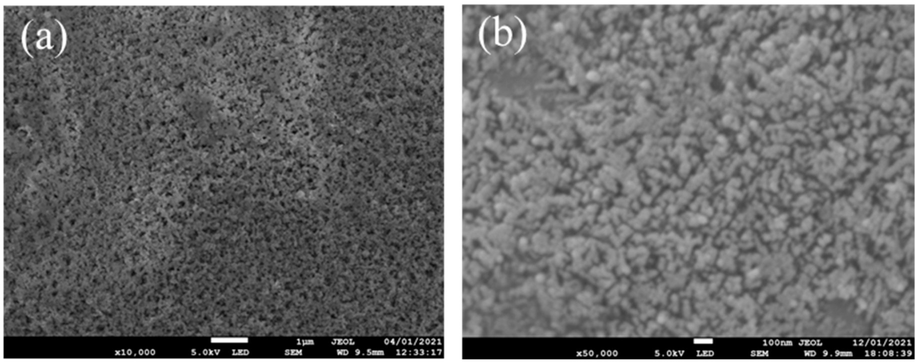

Figure S3. The SEM images of c-CuS (Prepared condition of c-CuS was the concentration of 1/2 mmol·L^{-1} and the Cu:S raw material ratio of 1:1 by using a single-jet liquid-phase sulfidation method)

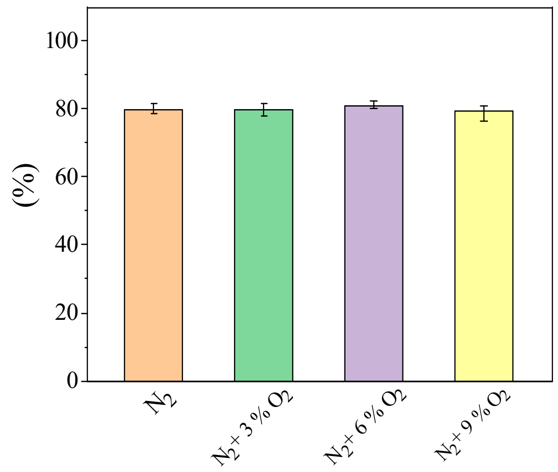

Figure S4. The effect of O_2.

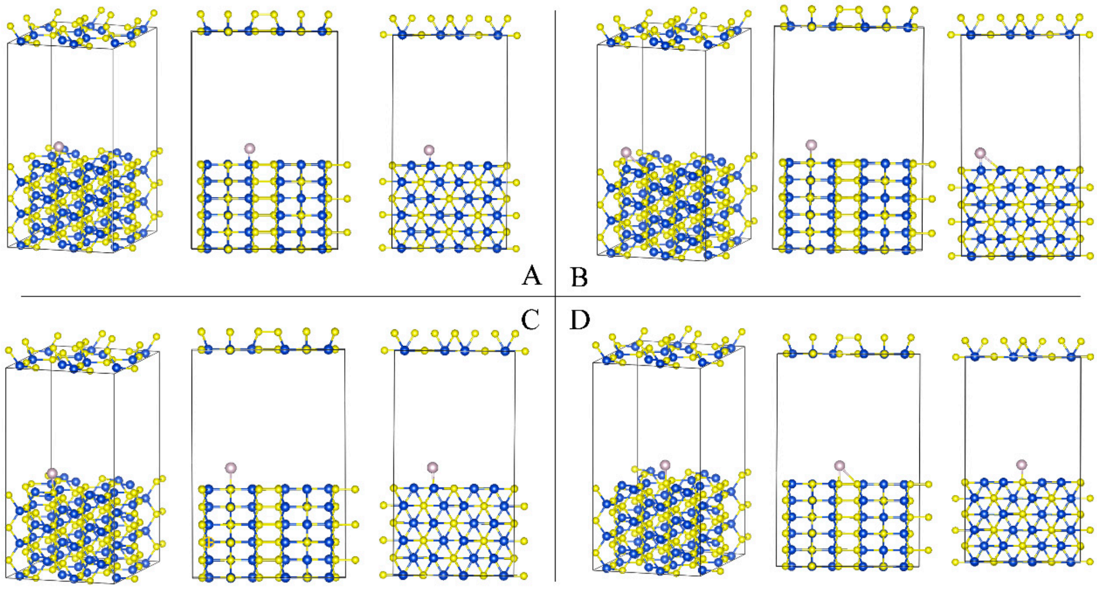

Figure S5. The structure of configurations A, B, C, and D after mercury adsorption.

TABLES:

Table S1. List of experimental conditions for Hg^0 capture.

Fig. number	*Solution volume*	*Solution pH*	*Solution temperature*	*Flue gas*
1a~1d	80 mL	3.0	20 °C	N_2
3a,3b	80 mL	3.0	20 °C	6% vol SO_2 +N_2
4a	80 mL	3.0	20 °C	N_2
4c	10 mL	3.0	20 °C	SFG+890μg·m^{-3} Hg^0

SFG: 6% vol O_2+6% vol SO_2+88% vol N_2.

Table S2. Hg^0 adsorption capacities and rates different sulfide sorbents.

Sorbents	*Hg^0 adsorption capacities (mg·g^{-1})*	*Average Hg^0 adsorption rates (μg·g^{-1}·min^{-1})*	*SO_2/O_2 vol percentage*	*Reference*
CA-CuS	736.49	306.88	6/6	This work
CuS@PUF	265.60	442.60	0/0	[1]
CuS	122.40	13.56	0/5	[2]
Fe_3O_4@CuS	88.70	13.25	0/0	[3]
ZnO@CuS	60.53	16.81	0/0	[4]
Nano-CuS	50.17	71.60	0/0	[5]
Co_3S_4	43.03	84.10	0/0	[6]
$FeS_{1.32}Se_{0.11}$	20.22	3.7	2.5/6	[7]
Cu_xS_y@AC	3.92	2.18	1.5/5	[8]
S/FeS_2	2.73	0.91	0/0	[9]
Nano-ZnS	0.49	0.26	0/0	[10]
Fe-ZnS	0.26	1.42	0/0	[11]

REFERENCES

[1] H. Li, Q. Yang, W. Qu, Y. Feng, L. Leng, J. Zhao, F. Meng, Z. Yang, J. Yang, Facile preparation of nanosized copper sulfide functionalized macroporous skeleton for efficient vapor-phase mercury sequestration, Chemical Engineering Journal 419 (2021) 129561.

[2] W. Liu, H. Xu, Y. Liao, Z. Quan, S. Li, S. Zhao, Z. Qu, N. Yan, Recyclable CuS sorbent with large mercury adsorption capacity in the presence of SO2 from non-ferrous metal smelting flue gas, Fuel 235 (2019) 847-854.

[3] Z. Yang, H. Li, C. Liao, J. Zhao, S. Feng, P. Li, X. Liu, J. Yang, K. Shih, Magnetic rattle-type Fe3O4@ CuS nanoparticles as recyclable sorbents for mercury capture from coal combustion flue gas, ACS Applied Nano Materials 1(9) (2018) 4726–4736.

[4] Q. Hong, H. Xu, J. Li, W. Huang, P. Liu, Z. Qu, N. Yan, Shell-thickness-induced spontaneous inward migration of mercury in porous ZnO@ CuS for gaseous mercury immobilization, Chemical Engineering Journal 420 (2021) 127592.

[5] Z. Yang, H. Li, S. Feng, P. Li, C. Liao, X. Liu, J. Zhao, J. Yang, P.-H. Lee, K. Shih, Multiform sulfur adsorption centers and copper-terminated active sites of nano-CuS for efficient elemental mercury capture from coal combustion flue gas, Langmuir 34(30) (2018) 8739-8749.

[6] Z. Quan, W. Huang, Y. Liao, W. Liu, H. Xu, N. Yan, Z. Qu, Study on the regenerable sulfur-resistant sorbent for mercury removal from nonferrous metal smelting flue gas, Fuel 241 (2019) 451-458.

[7] Z. Liu, Z. Li, X. Xie, S. Yang, J. Fei, Y. Li, Z. Xu, H. Liu, Development of recyclable iron sulfide/selenide microparticles with high performance for elemental mercury capture from smelting flue gas over a wide temperature range, Environmental science & technology 54(1) (2019) 604-612.

[8] S. Yang, D. Wang, H. Liu, C. Liu, X. Xie, Z. Xu, Z. Liu, Highly stable activated carbon composite material to selectively capture gas-phase elemental mercury from smelting flue gas: Copper polysulfide modification, Chemical Engineering Journal 358 (2019) 1235-1242.

[9] H. Li, W. Zhu, J. Yang, M. Zhang, J. Zhao, W. Qu, Sulfur abundant S/FeS2 for efficient removal of mercury from coal-fired power plants, Fuel 232 (2018) 476-484.

[10] H. Li, L. Zhu, J. Wang, L. Li, K. Shih, Development of nano-sulfide sorbent for efficient removal of elemental mercury from coal combustion fuel gas, Environmental science & technology 50(17) (2016) 9551-9557.

[11] Y. Xia, G. Wei, X. Liang, J. Zhu, H. Xian, X. Su, H. He, R. Zhu, Sequestration of Gaseous Hg0 by Sphalerite with Fe Substitution: Performance, Mechanism, and Structure–Activity Relationship, The Journal of Physical Chemistry C 123(5) (2019) 2828-2836.
